# Composite material consisting of microporous beta-TCP ceramic and alginate-dialdehyde-gelatin for controlled dual release of clindamycin and bone morphogenetic protein 2

**DOI:** 10.1007/s10856-023-06743-1

**Published:** 2023-07-27

**Authors:** Lucas Ritschl, Pia Schilling, Annette Wittmer, Marc Bohner, Anke Bernstein, Hagen Schmal, Michael Seidenstuecker

**Affiliations:** 1 G.E.R.N. Tissue Replacement, Regeneration & Neogenesis, Department of Orthopedics and Trauma Surgery, Medical Center Albert-Ludwigs-University of Freiburg, Faculty of Medicine, Albert-Ludwigs-University of Freiburg, Engesserstraße 4, 79108 Freiburg, Germany; 2 Medical Center Albert-Ludwigs-University of Freiburg, Institute of Microbiology and Hygiene, Hermann-Herder-Straße 11, 79104 Freiburg, Germany; 3 Robert Mathys Foundation, Bischmattstrasse 12, 2544 Bettlach, Switzerland; 4 Department of Orthopedics and Trauma Surgery, Medical Center Albert-Ludwigs-University of Freiburg, Hugstetter Straße 55, 79106 Freiburg, Germany

## Abstract

The aim of this study was to produce a composite of microporous β-TCP filled with alginate-gelatin crosslinked hydrogel, clindamycin and bone morphogenetic protein (BMP-2) to prolong the drug-release behaviour for up to 28 days. The most promising alginate-di-aldehyde(ADA)-gelatin gel for drug release from microcapsules was used to fill microporous β-TCP ceramics under directional flow in a special loading chamber. Dual release of clindamycin and BMP-2 was measured on days 1, 2, 3, 6, 9, 14, 21 and 28 by high performance liquid chromatography (HPLC) and enzyme-linked immunosorbent assay (ELISA). After release, the microbial efficacy of the clindamycin was checked and the biocompatibility of the composite was tested in cell culture. Clindamycin and the model substance FITC-protein A were released from microcapsules over 28 days. The clindamycin burst release was 43 ± 1%. For the loaded ceramics, a clindamycin release above the minimal inhibitory concentration (MIC) until day 9 and a burst release of 90.56 ± 2.96% were detected. BMP-2 was released from the loaded ceramics in low concentrations over 28 days. The release of active substances from β-TCP and hydrogel have already been extensively studied. Directional flow loading is a special procedure in which the ceramic could act as a stabilizer in the bone and, as a biodegradable system, enables a single-stage surgical procedure. Whether ADA-gelatin gel is suitable for this procedure as a more biodegradable alternative to pure alginate or whether a dual release is possible in this composite has not yet been investigated.

**Graphical Abstract:**

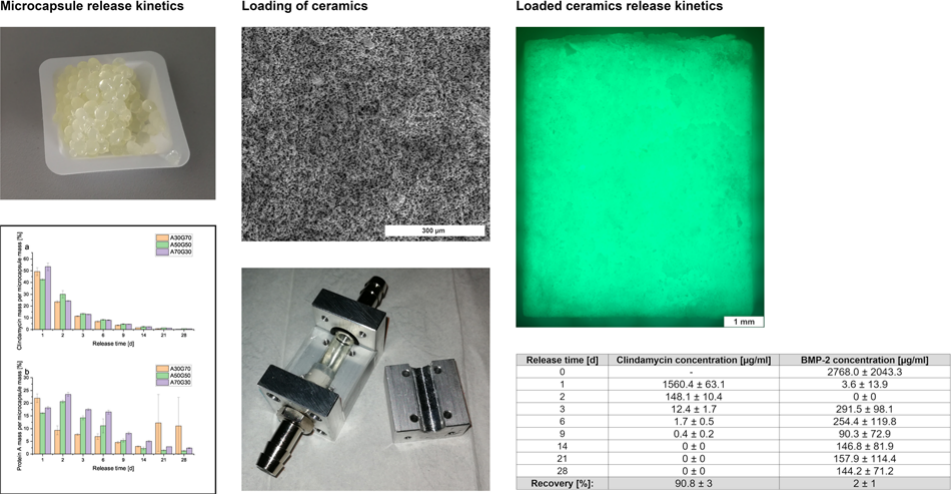

## Introduction

Osteomyelitis is an inflammation of the bone that usually affects the bone (osteitis), the bone marrow (osteomyelitis) and the periosteum (periostitis). The surrounding soft tissue may also be involved. It is triggered by an infection with various microorganisms and ultimately leads to the destruction of the bone. Aetiologically, a distinction is made between haematogenous, locally transmitted (per continuitatem), exogenous and specific osteomyelitis [1]. In paediatrics, haematogenous osteomyelitis affects the long bones in particular; in adults it manifests itself mainly as spondylitis [2]. The source of infection can be, for example, a trivial skin infection or a contamination during intravenous drug consumption, which is flushed haematogenously into the bone and triggers osteomyelitis there [3]. In the case of exogenous osteomyelitis, the post-traumatic form should be mentioned above all. Here, the pathogens can either be introduced through an open bone trauma or they enter the wound area through a surgical intervention [1]. A special form of the exogenous form is implant-associated osteomyelitis. It is characterised by biofilm formation of the colonising bacteria, in which the bacteria multiply less and are more resistant to antibiotics than outside the biofilm [4]. In the environment of an implant, atypical variants of the microorganisms can form, so-called small colony variants (SCV), which show a reduced division rate, atypical cell morphology, reduced pigment formation and lower metabolic performance [5]. Due to the low division rate and the sometimes-intracellular location, they are particularly resistant to antibiotics and can persist on the implant for years without causing an infection [5]. Under favourable conditions, they can cause a late infection by transforming into their original variant [6, 7]. Staphylococcus aureus is the most common pathogen of primary haematogenous and locally transmitted osteomyelitis [3]. In the case of implant-associated infection, the SCVs of the staphylococcus and coagulase-negative staphylococcus should be mentioned [7]. According to the duration of the infection or the histological form of inflammation, osteomyelitis can initially be divided into an acute and chronic form [6]. Osteomyelitis is considered chronic if the pathogen persists for more than 6 weeks, although the borderline can be blurred [1]. The appearance of sequesters on CT or MRI, on the other hand, is a sure criterion for a chronic infection [2]. Sequesters are necrotic pieces of bone that are rejected by healthy tissue and therefore prevent the infection from healing because they cannot be reached by the body’s immune system or by systemic antibiotics [8]. The typical therapy of a bone infection consists of the surgical removal of the infection site and the resulting determination of the pathogen [9]. Based on this, targeted systemic or local antibiotic therapies are applied. Sequestering and biofilm formation in combination with the poor bone penetrability of most antibiotics in the context of bone infections reduce the effectiveness of systemic antibiotic therapy. In contrast, local antibiotic applications can achieve higher levels of efficacy at the site of infection and reduce the systemic side effects of the therapy. Antibiotic-containing carrier systems are one possibility, which offer the advantage that the active substances can be released over a longer period of time and can thus fight the infection sufficiently even deep in the bone. Nowadays gentamycin-loaded non-biodegradable polyacrylmethacrylate (PMMA) bead chains (Septopal®) are introduced into the wound cavity and left there for 7–10 days [10]. These have the disadvantage that the chains have to be removed later in a second operation and a large part of the active substances remain in the PMMA [11]. Biodegradable alternatives are collagen fleeces loaded with gentamycin (Septocoll®) or collagen lyophilisates loaded with teicoplanin (Targobone®) [12]. Although one-stage surgical treatment is possible with these systems, the release of active substance lasts only a few days [13]. In vitro delayed drug release from a carrier system consisting of microporous β-TCP ceramics and alginate has already been described in the literature [14–16]. ADA-gelatin gel showed both better mechanical properties loaded in a TCP ceramic and a higher biodegradation than pure alginate [17]. As it had already been tested in relation to drug releases [18, 19], it was a promising hydrogel for special loading with directional flow. The possibility of dual drug release has not yet been investigated in this system, which could improve patient outcomes by combining anti-infective treatment, bone growth promoting therapy and better stability in the bone due to the TCP ceramic.

## Material and methods

### Preparing the ceramics

The ceramics were produced as previously described [20]. 80 g of a-tricaclium phosphate and 20 g of tricalcium phosphate (Art. No. 102143, Merck; mixture of an apatite and some calcium hydrogenphosphate) were mixed with 60.0 ± 0.2 g of a solution containing 0.2 M Na_2_HPO_4_ and 1% polyacrylic acid (Art. No. 81132, Fluka; Mw = 5.1 kDa). It was stirred and the paste was poured into plastic syringes with their tips removed (Ø = 23 mm). The paste hardened for 45 min, then was covered with 10 ml of PBS 7.4 solution (Art. No. P5368, Sigma) and incubated for 3 days at 60 °C. The samples (Ø = 23 mm; L = 70 mm) were dried at the same temperature and sintered at 1250 °C for 4 h. Heating and cooling took place at 1 °C/min. The cylinders were trimmed to a length of 25 mm and a diameter of 10 mm. At the end the samples were washed in ethanol to remove residual particles and calcined at 900 °C to burn off all organic residues. Before use the ceramics were shortened to cylinders with a length of 6 mm and washed again. The samples were sterilized at 200 °C for 4 h.

### Characterizing the ceramics

The structure and pore sizes were analyzed by ESEM (FEI Quanta 250 FEG, Hilsboro, USA), µCT (Scanco Micro-CT 50, Bruettisellen, Schweiz) and porosimetry (Porotec Pascal 140/440, Hofheim, Germany). For ESEM an acceleration voltage of 10 kV was used, the parameters of the µCT were 90 kV, 4 W, 44 µA at a resolution of 2 µm and an integration time of 5000 ms. The pore sizes were measured by Pascal 140 porosimeter in the range of 1000 µm to 1.4 µm with a pressure increase to 0.1 kPa and by Pascal 440 porosimeter in the range of 1.4 µm to 1.8 nm with a pressure increase to 400 MPa. The composition of the ceramics was defined via EDX (Philips ESEM XL 30 FEG, Amsterdam, Netherlands) and XRD (Bruker D8 Advance, Billerica, USA). EDX was performed with an acceleration voltage of 12 kV and a measure time of 100 s (live time corrected). Measurement conditions for XRD were Bragg-Brentano geometry, equipped with Cu anode and secondary graphite monochromator, scintillation counter, 40 kV/40 mA, 1°2-theta/min, step size 0.02°2theta.

### Preparing the hydrogel

Gelatin and alginate were sterilized by using low-temperature hydrogen peroxide gas plasma sterilization. The fabrication of alginate di-aldehyde (ADA) has been previously described in the literature [21]. 10 g alginate (Art. No. 71238, Sigma, St. Louis, USA) were dispersed in 50 ml ethanol and 3.21 g sodium periodate (Art. No. S1878, Sigma-Aldrich, St. Louis, USA) were dissolved in 50 ml distilled water. The alginate dispersion was stirred in the dark at 250 rpm at room temperature. The sodium periodate solution was slowly pipetted to the alginate dispersion. Stirring continuously in darkness, the reaction ran for six hours before it was stopped with 10 ml of ethylene glycol (Art. No. 324558, Sigma-Aldrich, St. Louis, USA). Stirring was continued for another half hour to ensure that the ethylene glycol was dispersed and the reaction stopped completely. The dispersion was then poured into a dialysis tube (Art. No. 132655, Spectrum Labs., Waltham, USA), which was completely submerged in a container of distilled water. Dialysis was carried out for one week, changing the water twice a day to remove sodium periodate, which would be toxic to cells. To check whether the water was periodate-free, 0.5 ml of it was mixed with 0.5 ml of 1%(w/v) silver nitrate solution (Art. No. 101512, Merck, Darmstadt, Germany). If periodate was still present, a yellowish-brown precipitate formed during the reaction. Finally, the ADA was frozen at −80 °C and then freeze-dried at −80 °C and 0.006 mbar. To prepare an ADA solution, the freeze-dried ADA flakes were dissolved again in PBS. To do this, PBS was pipetted onto the ADA flakes and the solution was stirred overnight at 100 rpm. Without this long stirring time, homogeneous ADA sol would not form. The gelatine was mixed with distilled water and left to swell at room temperature for 30 min. It was placed in the heating cabinet overnight at 37 °C and swirled repeatedly in between, until no more streaks formed. To produce ADA-gelatin gel, a 5%(w/v) solution of ADA and gelatin was prepared as described above. The gelatin solution was slowly added to the ADA solution with constant stirring at 200 rpm. This caused the gel to cloud, which gave a hint of the incipient cross-linking. The mixture was stirred for another 20 seconds and was then ready for further experiments.

### Characterizing the hydrogel

#### Gel permeation chromatography (GPC)

GPC analysis was carried out to determine the molar mass distribution and the mean molar masses of the used alginate, ADA and gelatin. 20 mg of the sample were dissolved in 10 ml of the eluent over two days at room temperature. Before measurement, the solutions were filtered through a PTFE filter membrane with a porosity of 1 µm. For calibration, several pullulan standards were used in the separation area of the column combination. The molar mass distributions and averages of the samples were calculated computer-aided by means of the so-called strip method using the pullulan calibration curve. The analysis conditions for GPC are shown in Table 1.

**Table 1:** Analysis conditions for GPC

Eluent:	0,02 M phosphate buffer, pH = 6,6 + 0,5 M NaCl aq.
**Columns:**	PSS Suprema, 10 µm, precolumn, ID 8,0 mm × 50 mm
	PSS Suprema, 10 µm, 100, ID 8,0 mm × 300 mm
	PSS Suprema, 10 µm, 3000, ID 8,0 mm × 300 mm
	PSS Suprema, 10 µm, 3000, ID 8,0 mm × 300 mm
**Column temperature:**	35 °C
**Pump:**	PSS SECcurity 1260 HPLC pump
**Flow rate:**	1,0 ml/min
**Injection system:**	PSS SECcurity 1260 autosampler
**Injection volume:**	50 µl
**Sample concentration:**	2 mg/ml
**Detector:**	PSS SECcurity 1260 RI-detector (differential refractometer)
**Analysis:**	PSS WinGPC UniChrom version 8.33

#### Rheology

The Malvern Kinexus lab+ KNX2110 rheometer (Malvern, UK) was used for the rheological investigations. The cone plate used (CP1/40 SR3033 SS) had a diameter of 40 mm and an angle of 1°. The distance to the fixed plate (PLS40 S2345 SS) was 23 µm. The measurements with frequency ramp were performed with a shear strain of 1% and a temperature of 25 °C in the range of 0.02 Hz–16 Hz. In order to determine the cross-linking point of the ADA-gelatin-hydrogel using the storage and loss module, a time ramp was also used, leaving all other parameters constant. These constant parameters were a temperature of 25 °C, a frequency of 1.6 Hz and a shear stress of 2 Pa. The frequency ramp was used to rheologically test 5%(w/v) ADA and 5%(w/v) gelatin brine, and the time ramp was used to measure the cross-linking points of ADA-gelatin gel with and without active ingredients.

#### Fourier-transform infrared spectroscopy (FTIR)

The Bruker Vector 22 (Billerica, USA) was used for the FTIR measurement. For this purpose, 5%(w/v) ADA solution in PBS and 5%(w/v) gelatin solution in distilled water were prepared. After the gelatin solution was slowly added to the ADA solution with constant stirring at 200 rpm, the gels were dried at 37 °C for one week in a heating cabinet. For the measurement, a resolution of 6 cm^−1^ was used. Each sample measurement was averaged over 100 individual measurements. The phase correction mode Mertz, the apodisation function Blackman-Harris 3-term and the zerofilling factor 2 were used.

### Loading the ceramics

The β-TCP ceramics were loaded using a procedure similar to that used by Seidenstuecker et al. [14]. The cut and cleaned ceramic scaffolds were clamped in a patented loading chamber (X5CrN18-10) where they were sealed with a silicone tube (stainless steel, inner diameter: 6 mm, outer diameter: 10 mm). Parafilm® M was used as a seal between the two halves of the chamber. The screwed loading chamber was connected on both sides with a two-way glass tap. On one side, the hydrogel was later filled in, and on the other side a fork was connected, linking the system to a desiccator and a vacuum pump. The desiccator served to stabilize the vacuum of 50 mbar generated by the vacuum pump (KNF Neuberger SC920, Freiburg, Germany) and was used as water trap. After switching on the pump, the two-way stopcock downstream of the loading chamber was opened to create a vacuum in the ceramic. After ten minutes, the gel was poured in and the front two-way stopcock was opened. After 30 min, the loading was stopped if no gel came out of the loading chamber. The ceramic was removed from the chamber and further cross-linked in CaCl_2_ solution to safely prevent the gel from escaping from the ceramic. With a gel that does not cross-link by itself, it was possible to load many ceramics in succession by simply exchanging the loading chamber together with the ceramic without having to make major changes to the experimental set-up. The cross-linking of the ADA-gelatin gel, on the other hand, made it necessary to be able to load several ceramics simultaneously in order to save time. Therefore, a parallel circuit consisting of six loading chambers was established, whereby the upstream two-way taps were dispensed with.

### Evaluating the loading of the ceramics

FITC-coupled protein A (Art. No. 101011, ThermoFischer, Waltham, USA) was used instead of BMP-2 and served as a model substance for BMP-2. This had the advantage for the evaluation of the loading that it could be detected by fluorescence microscopy. Accordingly, after loading, the ceramics were broken open longitudinally with a standard pair of pliers and the cross-section was recorded with the fluorescence microscope (Olympus BX51, Shinjuku, Japan). The FITC was made visible by an excitation of 485 nm and an emission of 514 nm. The loaded ceramic had a strong green glow, whereas an area that was not fully loaded was only very faintly green or completely dark. The individual images were stitched together using Microsoft Image Composite Editor (version 2.0.3.0). As the fluorescence decreased rapidly under illumination, rapid image acquisition was necessary.

### Setup of the drug-release experiment

#### Microcapsules

All steps were to be carried out protected from light, as both clindamycin and FITC were light-sensitive. Three different ADA/gelatine ratios were tested. A 300 mM calcium chloride solution was prepared containing 50 mg/ml clindamycin hydrochloride (Art. No. PHR1159, Sigma-Aldrich, St. Louis, USA) and 2.5% (v/v) FITC-protein-A solution. Gelatin/clindamycin was slowly added to ADA/FITC-protein A with constant stirring at 200 rpm and continued stirring for 20 s. 0.5 ml FITC-protein A solution and PBS were added to plasma sterilized ADA and stirred overnight at 100 rpm. 1 g clindamycin hydrochloride was dissolved in distilled water and plasmasterilised gelatin was added. It was allowed to swell for 30 min and placed in the warming oven at 37 °C overnight. In between, it was repeatedly swirled until no more streaks formed. The exact compositions of the gels with the different ratios of ADA and gelatin can be found in Table 2.

**Table 2:** Hydrogel composition for clindamycine-FITC-protein A microcapsules

Composition	ADA	PBS	Gelatin	Water
ADA30/FITC-PrA + gel.70/clindamycin	0.3 g	5.5 ml	0.7 g	14 ml
ADA70/FITC-PrA + gel.30/clindamycin	0.7 g	13.5 ml	0.3 g	6 ml
ADA50/FITC-PrA + gel.50/clindamycin	0.5 g	9.5 ml	0.5 g	10 ml

The gelatin-FITC-protein A solution was slowly added to the ADA solution with constant stirring at 200 rpm for 20 seconds. After 25 min (simulation of the loading time) 10 ml of the ADA/gelatin mixture were dropped into the prepared calcium chloride solution, slightly dried, weighed and distributed in approximately equal parts into four 5 ml vessels. All microcapsules distributed in vessels were completely covered with 2 ml of distilled water and then placed in the warming oven protected from light at 37 °C for 28 days. At day 1, 2, 3, 6, 9, 14, 21 and 28 after the experiment, a sample was taken from the water in the vessels and frozen at −20 °C. Afterwards, the water was completely replaced by new distilled water. At the end, all samples were thawed and sterile filtered with 0.2 µm disposable filters. The clindamycin content of the samples was determined via HPLC, the FITC-protein A content via fluorimetry.

#### Loaded ceramics

Clindamycin should be protected from light during loading. 1.5 g ADA from plasma sterilized alginate were dissolved in 600 µl BMP-2 stock solution (Art. No. 10426-HNAE, SinoBiological, Beijing, China; 0.5 g/l in distilled water with hydrochloric acid, pH 4, corresponding to 300 µg BMP-2) and 29.4 ml PBS. To obtain a homogeneous gel, the mixture was stirred overnight at 100 rpm. 3 g clindamycin hydrochloride were dissolved in 30 ml distilled water and 1.5 g sterilized gelatin was added. Gelatin/clindamycin was allowed to swell for 30 min and then placed in the warming cabinet overnight at 37 °C. It was swirled repeatedly until no streaks formed. A 600 mM CaCl_2_ solution was prepared and 50 mg/ml clindamycin hydrochloride was added. BMP-2 was omitted from the calcium chloride solution for cost reasons. The ADA portion was slowly added to the gelatin portion with constant stirring at 200 rpm and stirred further for 20 s. 18 ceramics were prepared, weighed and pressed into a silicone tube for sealing. Of the 18 ceramics, 15 were loaded with 6-fold parallel loading and the remaining three were carried unloaded as negative controls. After 30 min of loading, the ceramics were released from the chamber and from the silicone tube. The ceramics were freed from adhering gel residues and cross-linked for 24 h in 3 ml each of the prepared CaCl_2_ solution. The CaCl_2_ solutions were also kept as samples in order to be able to calculate the recovery. Between each loading, the tubing system and the loading chambers were cleaned to remove the cross-linked gel. The loaded ceramics were weighed again and transferred to 5 ml vials in the same way as the unloaded negative controls. Covered with 3 ml of distilled water, they were placed in the warming cabinet, protected from light, at 37 °C for 28 days. At day 1, 2, 3, 6, 9, 14, 21 and 28 after the experiment, the ceramics were transferred to a 5 ml vial with fresh distilled water and the old vial was deep frozen at −20 °C. At the end, all samples were thawed and sterile filtered with 0.2 µm disposable filters (Chromafil Xtra H-PTFE-20/25, Art. No. 729245, Macherey-Nagel, Düren, Germany). The antibiotic content of the samples was determined by HPLC, the BMP-2 content by ELISA.

### Determining concentration by HPLC, fluorimetry and ELISA

All samples were sterile filtered with a pore size of 0.2 µm before measurement. For clindamycin, HPLC (Shimadzu CBM-20A, CTO-20AC, DGU-20A5R, LC-20ADXR, Reservoir Tray, RF-20A, SIL-30AC, SPD-M20A IVDD, Kyōto, Japan; Macherey-Nagel precolumn EC 4/3 Nucleodur 300-5 C4ec, column EC 250/3 Nucleodur 300-5 C4ec, Düren, Germany) was carried out at a temperature of 25 °C, 10 min running time and 0.66 ml/min flow rate. ACN and 25.08 mM Na_2_HPO_4_ (pH 3,5; adjusted with phosphoric acid) in relation 29:71 was used as a mobile phase. The AUC was measured at wavelength of 193 nm and a retention time of 3.49 ± 0.06 min. The PerkinElmer EnSight Multimode Plate Reader (Waltham, USA) was used for fluorimetry. For the measurement, samples with a volume of 200 µl were pipetted into black 96-well plates. The concentration of FITC-protein A was determined at an excitation wavelength of 490 nm and an emission wavelength of 525 nm. ELISA was performed with the Human BMP-2 ELISA Kit from Sino Biological (Art. No. KIT10426, Beijing, China) according to the manufacturer’s instructions [22]. A 96-well plate coated with capture antibody was already part of the kit. After rinsing three times with 300 µl wash buffer, 100 µl of each release sample was pipetted into the wells. In addition, a BMP-2 standard (0–2500 pg/ml) was prepared and treated like the samples. The samples and the standard were completely pipetted within 15 min and incubated for 2 h at room temperature. The wells were then rinsed three times again, 100 µl of the detection antibody was added and incubated for 1 h at room temperature. The wells were rinsed three times and 200 µl of the substrate solution was pipetted into the wells. After 20 min incubation at room temperature in darkness, the colour reaction was terminated with 50 µl of stop solution and the absorbance was determined at 450 nm.

### Determining the minimal inhibitory concentration

To prove that clindamycin was still microbially effective after 28 days, its minimum inhibitory concentration was determined. This was done according to ISO Standard 20776-1 and EUCAST [23]. Samples from release days 1, 2, 3, 6, 9, 14, 21 and 28 of clindamycin-BMP-release were tested. The antibiotic concentrations determined by HPLC made it possible to test a dilution series with fixed concentrations for each release day in addition to the original concentration. The initial concentration was set with distilled water, the following dilution was carried out with Müller-Hinton-Bouillon (MHB) (BBL Mueller Hinton II Broth (Cation-Adjusted), Art. No. 298268, BD Biosciences, Franklin Lakes, USA). In addition to the samples, growth controls were prepared with 100 µl MHB plus 5 µl bacterial suspension and blank values with pure MHB. In addition, samples of ceramics with ADA-gelatine loading without active ingredients were tested to determine whether the low proportion of MHB in the initial preparation had a negative effect on bacterial growth and whether the ADA-gelatine gel alone had an antimicrobial effect. For comparison, these samples were diluted 1:1 with MHB. Two identical preparations were made from each sample. For the experiments, the standard strain of Staphylococcus aureus ATCC 29213 was pre-incubated on CBA-plates (Colombia-Blood-Agar; Oxoid™, Thermo Scientific, Waltham, USA) for 24 h and adjusted to McFarland 0.5 with physiological NaCl solution [24]. This corresponded to a bacterial count of 1 x 10^8^ per millilitre. Now this was diluted with MHB 1:10 to a concentration of 1 x 10^7^ ml^−1^. This resulted in the required bacterial count of 5 x 10^5^ ml^−1^ when 5 µl of this bacterial suspension was mixed with 100 µl of the respective sample for the MIC test. For the inoculum control, 10 µl from a growth control were diluted 1:1000 in MHB and 100 µl were plated out on Colombia-blood-agar beforehand. Thus, 50 CFU had to grow on the plate for the required bacterial count of 5 x 10^5^ ml^−1^. After incubation at 35 °C for 18 ± 2 h, bacterial growth was monitored and the MIC determined.

### Release kinetics

The release kinetics were determined by using the model of Ritger and Peppas [25]. They used the formula

$$\frac{{M_t}}{{M_\infty }} = kt^n$$

with M_t_ as the mass of released drug at time t, M_∞_ as the mass of released drug as time approaches infinity, k as a constant incorporating the characteristics of the macromolecular network system plus the drug and n as a diffusional coefficient which indicates the transport mechanism [25]. The exponent n is also dependent on the geometrics of the sample. Fickian release is described at *n* = 0.43 for spherical samples and *n* = 0.45 for cylindrical samples [25].

### Cell culture experiments

In order to sterilely load the ceramics for cell culture, a procedure with undirected vacuum was used. For this purpose, all materials used, such as tubes, desiccator, etc., were disinfected with ethanol. The plasma sterilised gels with the active substances were dissolved under a fume cupboard, the gelatin part was then poured into the ADA part, briefly vortexed and the ADA-gelatin gel was filled into a sterile beaker. The cleaned heat-sterilized ceramic scaffolds were sunk into this gel and the beaker was placed in the desiccator without a lid. At a pressure of 50 mbar over a period of 5 min, small bubbles were seen to rise from the ceramic. The vacuum was then released, and the desiccator opened. The ceramics remained in the ADA-gelatin gel for 20 min at normal pressure, were taken out with tweezers, lightly cleaned of any gel residues adhering to the outside and placed in a 600 mM calcium chloride solution for 24 h. Three 2 mm long β-TCP ceramics were prepared for clindamycin and BMP-2. In addition, three samples were prepared with the ADA-gelatin ratio 50:50 without active substances and an empty ceramic was treated as a positive control and a blank for each day. All ceramics were pressed into a 12 mm long silicone tube with an inner diameter of 6 mm and an outer diameter of 10 mm, so that the ceramic flattened one end of the tube. This was the same type of tubing used for sealing during directional loading. This served to keep the cell suspension on the ceramics so that the cells were given the opportunity to grow appropriately. For this purpose, cells were added to each ceramic in a medium volume of 200 µl. The cells had 5 h to settle on the ceramics at an incubation of 37 °C and 5% CO_2_. The ceramics were carefully cut out of the silicone tube with a scalpel. Care had to be taken not to contaminate the experiment and not to accidentally place the ceramics with the cell-covered top side down. The wells were filled with medium until the ceramics were completely covered. Live-dead assay (PromoCell Live/Dead Cell Staining Kit II, Art. No. PK-CA707-30002, Heidelberg, Germany), WST assay (Roche Cell Proliferation Reagent WST-1, Art. No. 11644807001, Basel, Switzerland) and LDH assay (Roche Cytotoxicity Detection Kit (LDH), Art. No. 11644793001, Basel, Switzerland) were performed according to the manufacturer’s instructions [26–28]. For live-dead assay 20k cells per sample, for WST and LDH assay 50 k cells per sample were used.

### Statistics

Data was presented as average ± standard deviation and analysed using one-way analysis of variance (ANOVA). The comparison of averages was done according to Fisher LSD. The statistical significance level was set at *p* < 0.05. Calculations were performed using OriginPro 9.7 (OriginLabs, Northampton, MA, USA).

## Results

### Characterizing the ceramics

**Fig. 1:**
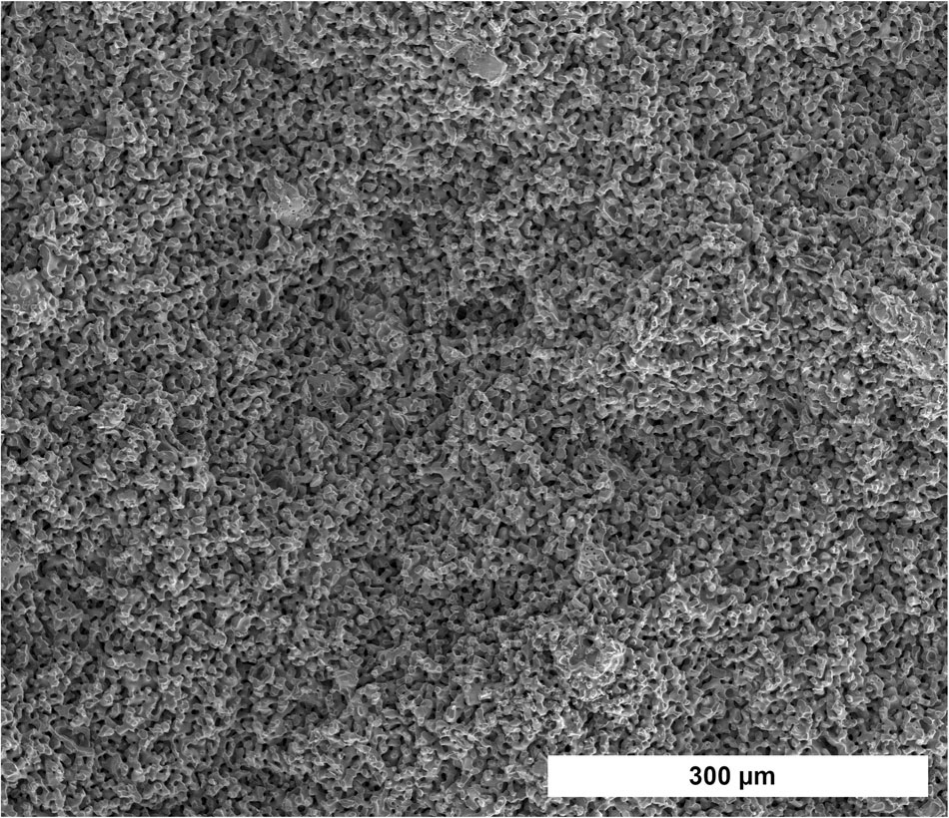
ESEM image of the RMS ceramic (FEI Quanta 250 FEG, Hilsboro, USA; acceleration voltage 10 kV; Large Field Detector; pressure 100 Pa; WD 9.2 mm; HFW 746 µm; 400x magnification)

The ESEM images show a uniform microporous structure of the RMS ceramic (Fig. 1).

**Fig. 2:**
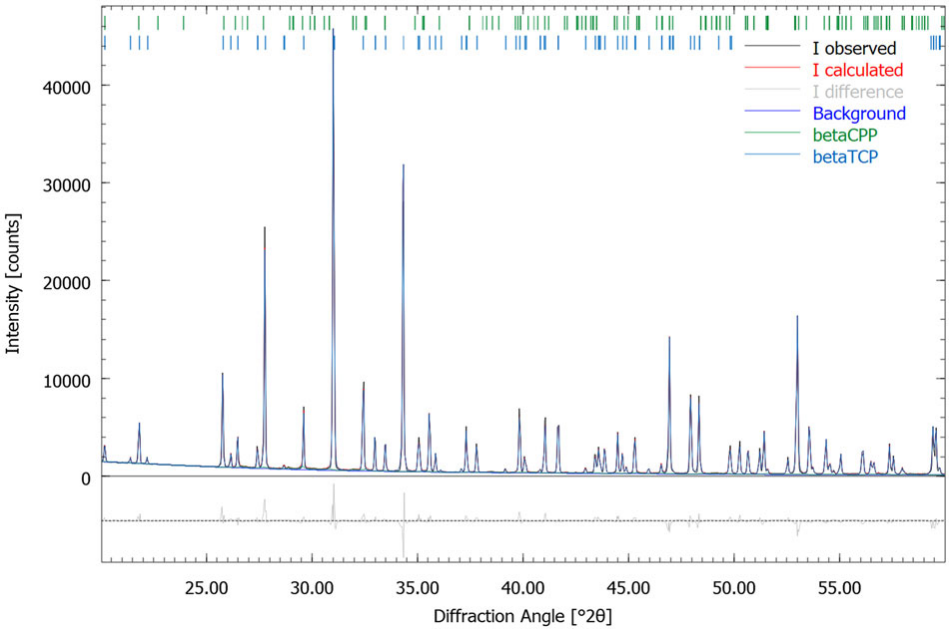
XRD spectrum of the RMS ceramic (Bruker D8 Advance, Billerica, USA; Bragg-Brentano geometry; Cu anode; secondary graphite monochromator; scintillation counter; 40 kV/40 mA; 1°2-theta/min; step size 0.02°2theta)

In EDX 19.1 at% calcium and 13.3 at% phospor were found in the RMS ceramic. With a Ca/P ratio of 1.44 EDX gave an indication that the RMS ceramic consisted of β-TCP (Ca/P ratio 1.5) [29]. XRD confirmed the β-TCP consistence of the RMS ceramic. Traces of β-CPP could be detected, shown in Fig. 2.

**Fig. 3:**
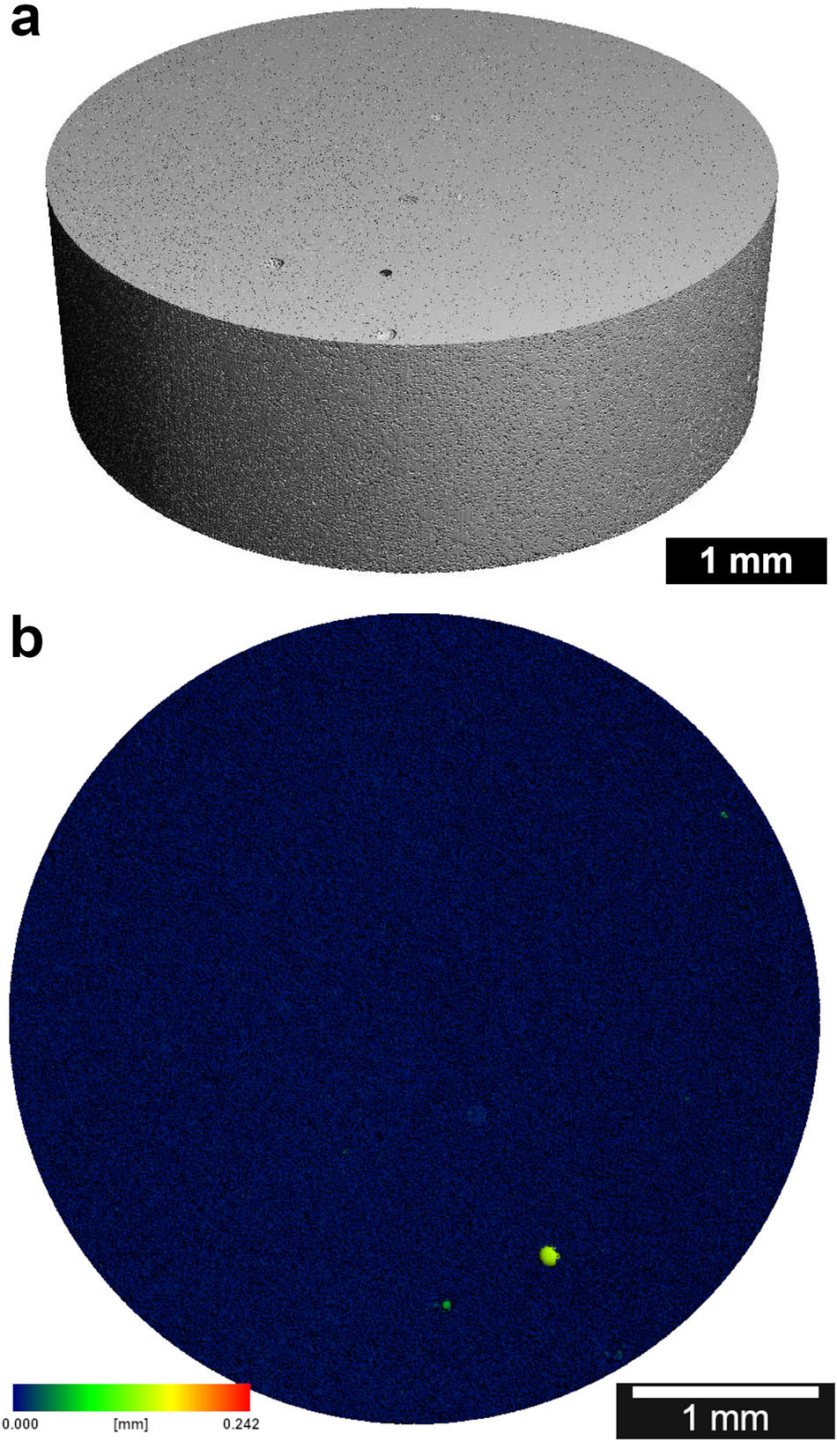
Micro-CT reconstruction of the ceramic (**a**) and of the porosity of the ceramic (**b**); 90 kV, 4 W, 44 µA, resolution 2 µm, integration time 5000 ms (Scanco Micro-CT 50, Bruettisellen, Switzerland)

In micro-CT, the RMS ceramic had small pore sizes with occasional larger pores, shown in Fig. 3.

**Fig. 4:**
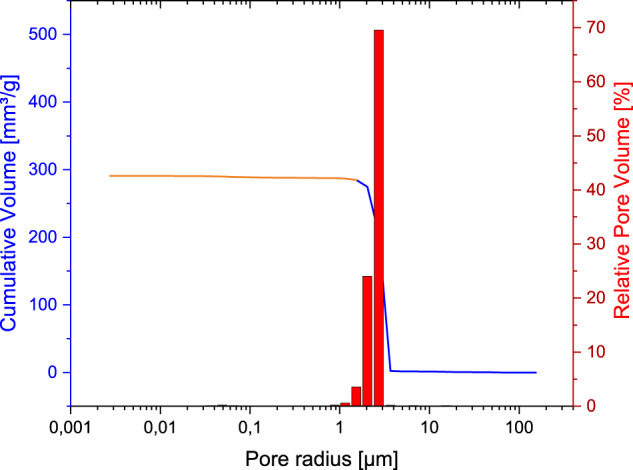
Pore size distribution of the RMS ceramic, measurements of the Pascal-140 porosimeter (1000 µm–1.4 µm; pressure increase to 0.1 kPa) in blue and the Pascal-440 porosimeter (1.4 µm–1.8 nm; pressure increase to 400 MPa) in orange (Porotec Pascal 140/440, Hofheim, Germany)

The RMS ceramic showed a total porosity of 48% and an average pore radius of 2.59 µm in porosimetry. The pore size distribution showed that 95% of the pore sizes were between 1 and 3 µm and is shown in Fig. 4. Here, the use of the two different porosimeters was taken into account in terms of colour when displaying the cumulative pore volumes. The low pressure range of the Pascal-140 porosimeter was shown in blue and the high pressure range of the Pascal-440 porosimeter in orange.

### Characterizing the hydrogel

#### Gel permeation chromatography (GPC)

Alginate’s and gelatin’s mean molar masses were just slightly changed by plasma sterilization showing plasma sterilization as a suitable procedure to sterilize these hydrogels. ADA had a clearly lower molar mass than alginate. The results of GPC are shown in Table 3.

**Table 3:** Mean molar mass of alginate, gelatin and ADA; changed by plasma sterilization

Sample	M_n_ [kDa]	M_w_ [kDa]	M_z_ [kDa]	PDI (= M_w_/M_n_)
Alginate before plasma sterilization	198	729	1440	3.68
Alginate after plasma sterilization	201	767	1530	3.81
ADA from plasma sterilized alginate	55	298	1160	5.38
Gelatin before plasma sterilization	16.3	114	254	6.99
Gelatin after plasma sterilization	16	105	231	6.61

#### Rheology

**Fig. 5:**
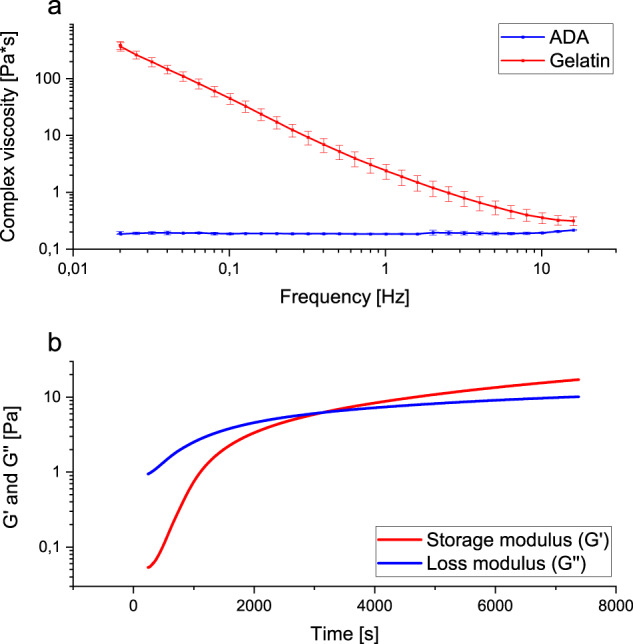
**a** Complex viscosity of ADA and gelatin depending on the frequency; shear strain 1%; temperature 25 °C; 0.02–16 Hz; cone plate (CP1/40 SR3033 SS) with diameter 40 mm and angle 1°; distance to fixed plate (PLS40 S2345 SS) 23 µm (Malvern Kinexus lab+ KNX2110 rheometer, Malvern, UK). **b** Storage and loss modulus of ADA-gelatin depending on the time; temperature 25 °C; frequency 1.6 Hz; shear stress 2 Pa; cone plate (CP1/40 SR3033 SS) with diameter 40 mm and angle 1°; distance to fixed plate (PLS40 S2345 SS) 23 µm (Malvern Kinexus lab+ KNX2110 rheometer, Malvern, UK)

Complex viscosity of 5% (w/v) ADA and 5% (w/v) gelatin was shown in Fig. 5a. Sterile filtration with a pore size of 0.45 µm proved to be a gentle sterilisation process for ADA, as it only minimally changed its rheological properties. The ADA-gelatin measurements showed irregularities in the cross-linking behaviour. In one of the five measurements, the ADA-gelatin mixture did not crosslink at all within 2 h. In the other four measurements, the sol/gel transition points could be determined via the intersections of storage and loss modulus. The times until cross-linking were 43, 52, 69, and 113 min. An exemplary measurement was shown in Fig. 5b. Here, the time after the start of cross-linking until the time when the measurement could be started was added. This undocumented time at the beginning of the cross-linking was between 3 and 4 min. When the ADA-gelatin sols contained additional clindamycin and BMP-2, the storage and loss moduli only approached each other, but did not cross over even after two hours. This circumstance made further cross-linking with calcium ions indispensable. Although the rheological measurements did not completely coincide with the observations during the loadings, since gels with clindamycin and BMP-2 could be cross-linked here, they were by far not as frequent as with ADA-gelatin sols without active ingredients.

#### Fourier-transform infrared spectroscopy (FTIR)

**Fig. 6:**
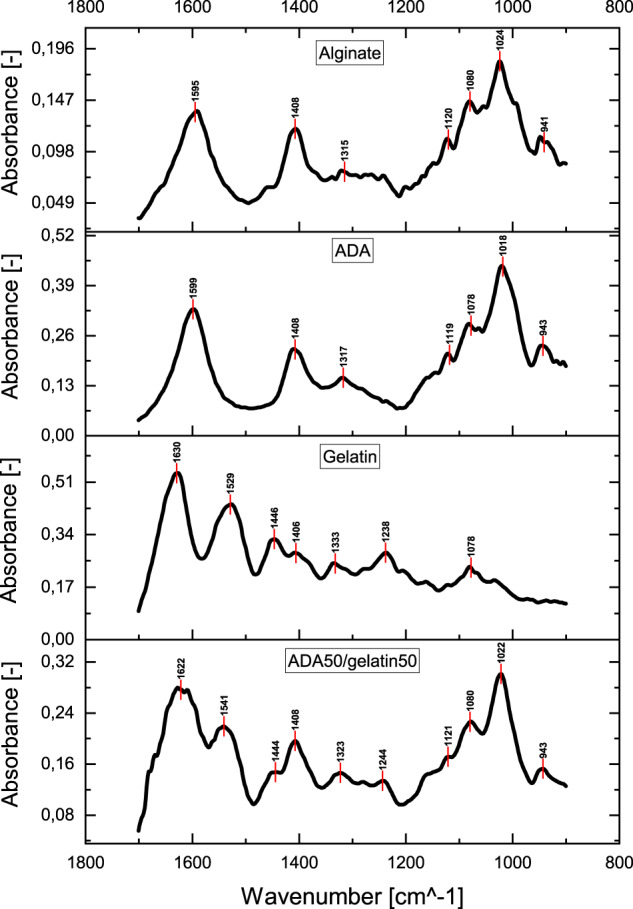
FTIR spectra of alginate, ADA, gelatin and ADA50/gelatin50 in comparison (Bruker Vector 22, Billerica, USA; resolution 6 cm^−1^; phase correction mode Mertz; apodisation function Blackman-Harris 3-term; zerofilling factor 2)

FTIR was used to evaluate the cross-linking of ADA and gelatin. Both overlaps and shifts of the peaks were observed (see Fig. 6). For example, ADA had a peak at 1599 cm^−1^. Gelatin had peaks at 1630 cm^−1^ and 1529 cm^−1^ that matched C = O of the primary amide and N-H of the secondary amide of gelatin, respectively. The ADA-gelatin gel, on the other hand, had characteristic peaks at 1622 cm^−1^ and 1541 cm^−1^ indicating ν(C = O) and thus the Schiff’s base reaction. The peak at 1622 cm^−1^ was probably an overlap with the peak at 1630 cm^−1^, which is characteristic for gelatin. The band at 1529 cm^−1^ of the gelatin became smaller with increasing ADA content and shifted to a higher region, which also spoke for the Schiff’s base reaction [21, 30–32].

### Evaluating the loading of the ceramics

**Fig. 7:**
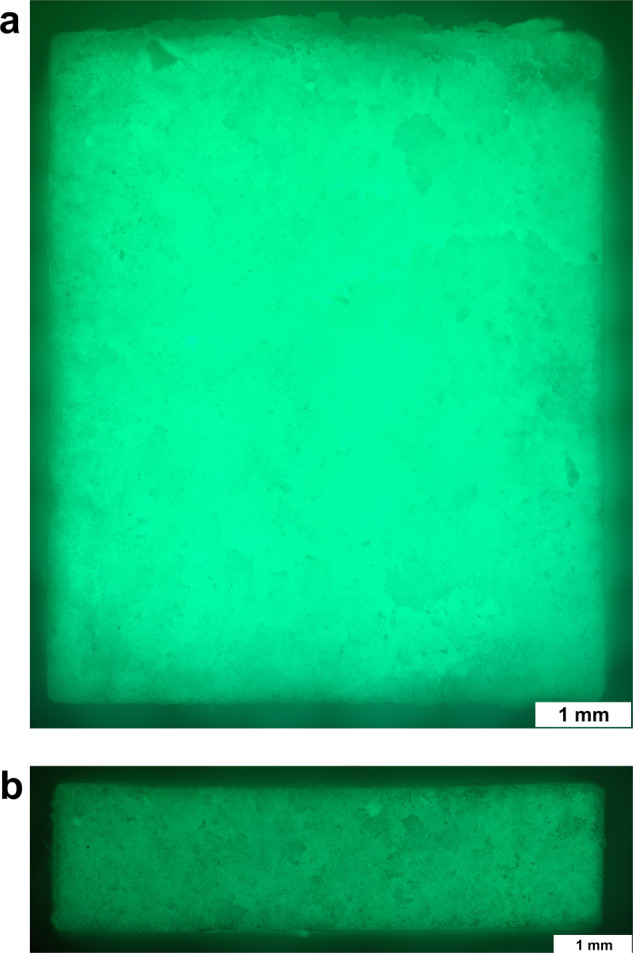
**a** Ceramic cross-section under the fluorescence microscope after directional loading with FITC-protein A gel. **b** Ceramic cross-section under the fluorescence microscope after undirectional loading with FITC-protein A gel. Microscope: Olympus BX51, Shinjuku, Japan

To show the successful directional loading of the ceramics, FITC-protein A was added to the gel and the ceramic cross-section was displayed under the fluorescence microscope. The ceramics were completely loaded (see Fig. 7a). Even with the sterile loading technique with undirected vacuum, the ceramic cross-sections were homogeneously fluorescent (see Fig. 7b). When gel-loaded with clindamycin/BMP-2, the ceramics gained an average of 114 ± 4 mg in weight due to the loading.

### Drug release

#### Microcapsules

In the following, the results of the release from the microcapsules are presented as active substance mass in relation to the mass of microcapsules. This was necessary because the microcapsules were not arbitrarily small. Therefore, they could not be distributed exactly equally among the different release approaches. Accordingly, a normalisation to the mass of microcapsules contained in the test batch was necessary for the averaging. The average mass of all microcapsules in a release vessel was 2.5 ± 0.3 g.

**Fig. 8:**
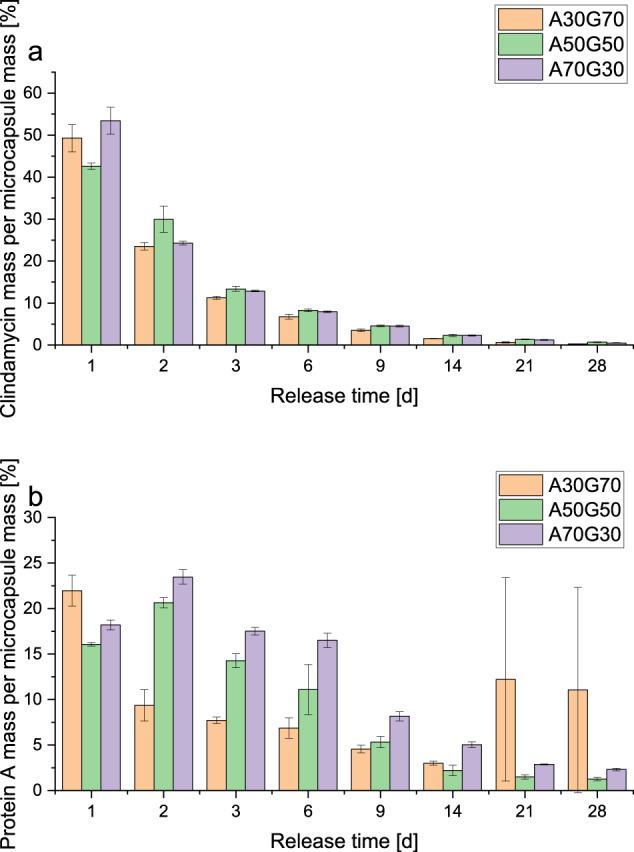
**a** Clindamycin release from the microcapsules in relation to the total amount of clindamycin. **b** Protein A release from the clindamycin/FITC-protein A microcapsules in relation to the total amount of protein A. A: ADA, G: gelatin, number: percentage in the gel

Figure 8a shows the clindamycin release from the different microcapsules. A50G50 had a statistically significantly lower burst release than the other samples. Averaged over all microcapsules, the clindamycin recovery was 105 ± 5%. The protein A release is shown in Fig. 8b. There was a statistically significant difference between all the different ADA/gelatin ratios. A50G50 released the least protein A on the first day. On days 21 and 28, more protein A was detected in A30G70 compared to the other samples and days. The individual samples of this release differed greatly from each other, which resulted in the high standard deviation. The cumulative protein A release resulted in an average protein A recovery of 77 ± 15%.

#### Loaded ceramics

Due to the absence of BMP in the calcium chloride solution, the BMP release resulted in a measurement at day 0 that mapped the concentration in the calcium chloride solution. This was necessary to be able to calculate the recovery correctly later.

The burst release of clindamycin was 90.6 ± 3% in relation to the detected clindamycin mass. By day 9, the concentration was above the MIC of clindamycin, from day 14, clindamycin could no longer be detected. The slightly delayed BMP release was noticeable, which only started from day 3 and was then rather constant compared to the antibiotic. Overall, only a small fraction of the BMP theoretically contained in the loaded ceramics could be recovered in this experiment (see Table 4).

**Table 4:** Released clindamycin and BMP-2 concentration after clindamycin/BMP-2 loading

Release time [d]	Clindamycin concentration [µg/ml]	BMP-2 concentration [µg/ml]
0	-	2768.0 ± 2043.3
1	1560.4 ± 63.1	3.6 ± 13.9
2	148.1 ± 10.4	0 ± 0
3	12.4 ± 1.7	291.5 ± 98.1
6	1.7 ± 0.5	254.4 ± 119.8
9	0.4 ± 0.2	90.3 ± 72.9
14	0 ± 0	146.8 ± 81.9
21	0 ± 0	157.9 ± 114.4
28	0 ± 0	144.2 ± 71.2
**Recovery [%]:**	90.8 ± 3	2 ± 1

#### Model of release kinetics

**Fig. 9:**
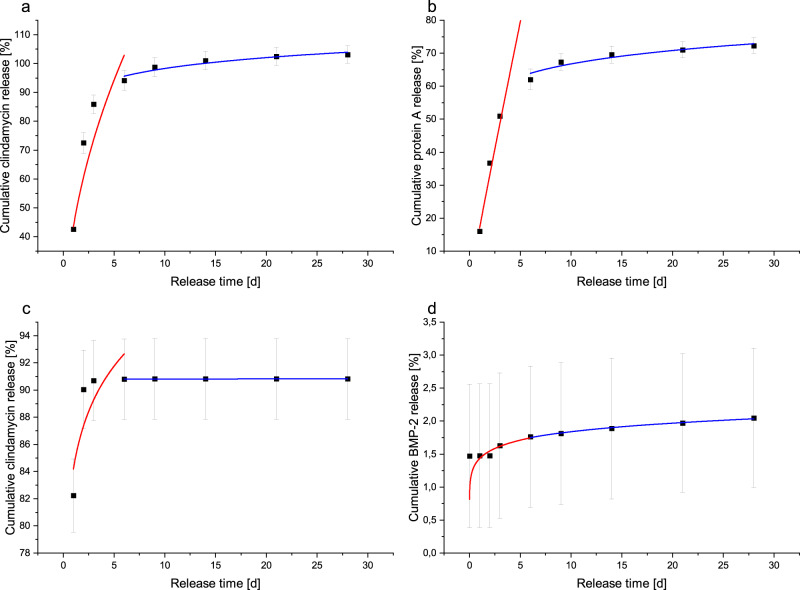
Fitting of release according to Ritger/Peppas till day 6 in red and from day 6 in blue. **a** Clindamycin release from A50G50 microcapsules. **b** Protein A release from A50G50 microcapsules. **c** Clindamycin release from loaded ceramics. **d** BMP-2 release from loaded ceramics

The analysis of the release kinetics according to Ritger-Peppas showed low values for n for the loaded ceramics (see Table 5). This corresponded to anomalous release kinetics. For the microcapsules, n was close to 0.43 with a value of 0.48 at the beginning of clindamycin release (see Table 5), which corresponds to a Fickian diffusion for spherical samples. In the case of protein A release, there was even an almost zero-order release at the beginning with an n of 0.96. The cumulative release curves for the A50G50 microcapsules confirm this result with an almost linear course at the beginning (see Fig. 9). The beginning and the end of the release were considered separately, as the analysis according to Ritger-Peppas had the highest significance in the first 20% of the release.

**Table 5:** Overview of used parameters for fitting according to Ritger/Peppas (y = kxn), fitting till day 6 labelled with start and from day 6 with end, R: Pearson correlation coefficient

	Clindamycin						Protein					
	n_start_	k_start_	R^2^_start_	n_end_	k_end_	R^2^_end_	n_start_	k_start_	R^2^_start_	n_end_	k_end_	R^2^_end_
**Microcapsules**	0.48	43.48	0.88	0.05	86.77	0.88	0.96	17.06	0.9	0.09	54.86	0.88
**Loaded ceramics**	0.05	84.19	0.56	0.001	90.78	0.37	0.11	1.43	0.81	0.1	1.47	0.98

### Minimal inhibitory concentration

By means of the MIC test, the bacteriostatic efficacy of clindamycin could be shown after the different release days. The MIC for clindamycin/BMP release ranged from 0.125 to 0.8 µg/ml (see Table 6). On release days 1 to 3 the MIC was constant at 0.125 µg/ml, on day 6 it increased to 0.203 µg/ml and on day 9–0.8 µg/ml. For days 14 to 28, neither HPLC nor microbiological testing could detect the antibiotic. The inoculum control showed a bacterial concentration of 7 x 10^4^ ml^−1^.

**Table 6:** MIC test of clindamycin/BMP release (the bold values refer to the concentration in which the following two measurements were tested, c: Clindamycin concentration, T: day, P: sample, (−): no growth, (+): growth, (++): strong growth, (+++): very strong growth, /: no measurement, GC: growth control, MIC [mg/l])

Sample	1	2	3	4	5	6	7	8	9	10	11	12	MIC
**c [mg/l]**	**1549.10**	**4**	**2**	**1**	**0.5**	**0.25**	**0.125**	**0.063**	**0.031**	**0.016**	**GC**	**Blank**	**MIC**
T1P1	-	-	-	-	-	-	-	++	+++	+++	+++	-	0.125
T1P2	-	-	-	-	-	-	-	++	+++	+++	+++	-	0.125
**c [mg/l]**	**148.49**	**4**	**2**	**1**	**0.5**	**0.25**	**0.125**	**0.063**	**0.031**	**0.016**	**GC**	**Blank**	**MIC**
T2P1	-	-	-	-	-	-	-	++	+++	+++	+++	-	0.125
T2P2	-	-	-	-	-	-	-	++	+++	+++	+++	-	0.125
**c [mg/l]**	**13.96**	**4**	**2**	**1**	**0.5**	**0.25**	**0.125**	**0.063**	**0.031**	**0.016**	**GC**	**Blank**	**MIC**
T3P1	-	-	-	-	-	-	-	++	+++	+++	+++	-	0.125
T3P2	-	-	-	-	-	-	-	++	+++	+++	+++	-	0.125
**c [mg/l]**	**1.62**	**-**	**0.81**	**0.405**	**0.203**	**0.101**	**0.051**	**0.025**	**0.013**	**0.006**	**GC**	**Blank**	**MIC**
T6P1	-	/	-	-	-	++	+++	+++	+++	+++	+++	-	0.203
T6P2	-	/	-	-	-	++	+++	+++	+++	+++	+++	-	0.203
**c [mg/l]**	**0.8**	**-**	**0.4**	**0.2**	**0.1**	**0.05**	**0.025**	**0.013**	**0.006**	**0.003**	**GC**	**Blank**	**MIC**
T9P1	-	/	+	++	+++	+++	+++	+++	+++	+++	+++	-	0.8
T9P2	-	/	+	++	+++	+++	+++	+++	+++	+++	+++	-	0.8
**c [mg/l]**	**0**	**-**	**0**	**0**	**0**	**0**	**0**	**0**	**0**	**0**	**GC**	**Blank**	**MIC**
T14P1	+	/	+++	+++	+++	+++	+++	+++	+++	+++	+++	-	> 0
T14P2	+	/	+++	+++	+++	+++	+++	+++	+++	+++	+++	-	> 0
**c [mg/l]**	**0**	**-**	**0**	**0**	**0**	**0**	**0**	**0**	**0**	**0**	**GC**	**Blank**	**MIC**
T21P1	++	/	+++	+++	+++	+++	+++	+++	+++	+++	+++	-	> 0
T21P2	++	/	+++	+++	+++	+++	+++	+++	+++	+++	+++	-	> 0
**c [mg/l]**	**0**	**-**	**0**	**0**	**0**	**0**	**0**	**0**	**0**	**0**	**GC**	**Blank**	**MIC**
T28P1	++	/	+++	+++	+++	+++	+++	+++	+++	+++	+++	-	> 0
T28P2	++	/	+++	+++	+++	+++	+++	+++	+++	+++	+++	-	> 0

### Cell culture experiments

**Fig. 10:**
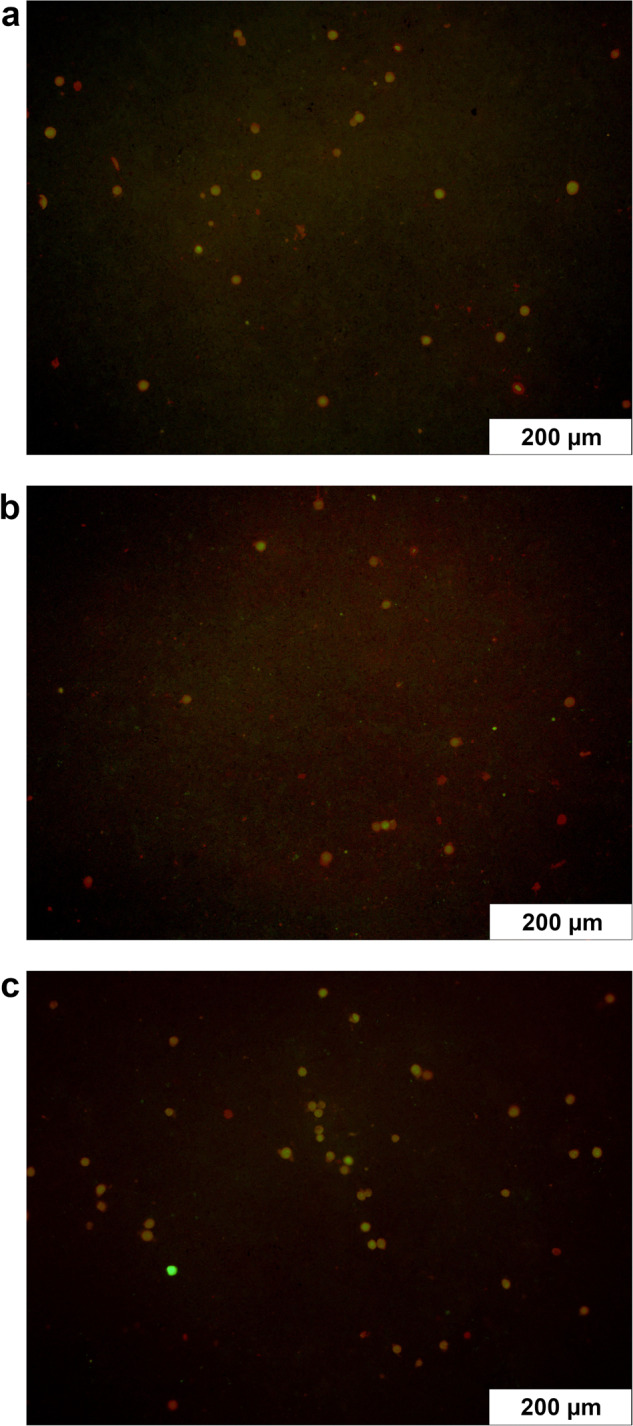
Comparative cell growth on clindamycin-BMP-composite after 3 (**a**), 7 (**b**) and 10 (**c**) days; living cells in green, dead cells in red; images taken with 10x magnification (Olympus BX51, Tokio, Japan)

The proportions of dead, living and intermediate cells on the different samples could be shown in the live dead assay (see Fig. 10). Intermediate cells were those that could be stained with both EthD-III and calcein. The experiments showed a high percentage of live cells on the ceramics loaded with ADA-gelatin gel without active ingredients. In addition, cell viability was high on the unloaded ceramics. On the clindamycin-loaded ceramics, a significantly lower proportion of live cells and a higher proportion of intermediate cells were observed (see Table 7).

**Table 7:** Mean and standard deviation of absolute and relative cell counts of different samples in live-dead assay (ADA-gelat. w/o a.i. = ADA-gelatin without active ingredients)

	Absolute cell count [cells/cm^2^]				Relative cell count [%]		
	Alive	Intermediate	Dead	Overall	Alive	Intermediate	Dead
**Day 3**							
Clindamycin/BMP	44 ± 41	6331 ± 3315	3165 ± 1585	9540 ± 4831	0.5 ± 0.4	66 ± 35	33 ± 17
ADA-gelat. w/o a.i.	60230 ± 19493	0 ± 0	2198 ± 1206	62428 ± 19938	96 ± 31	0 ± 0	4 ± 2
Empty ceramic	47701 ± 7063	0 ± 0	2022 ± 597	49723 ± 7637	96 ± 14	0 ± 0	4 ± 1
**Day 7**							
Clindamycin/BMP	88 ± 104	1934 ± 2025	1539 ± 795	3561 ± 2709	2 ± 3	54 ± 57	43 ± 22
ADA-gelat. w/o a.i.	27170 ± 10311	0 ± 0	5583 ± 1572	32753 ± 9959	83 ± 31	0 ± 0	17 ± 5
Empty ceramic	65506 ± 7936	0 ± 0	4528 ± 1033	70034 ± 8795	94 ± 11	0 ± 0	6 ± 1
**Day 10**							
Clindamycin/BMP	132 ± 120	8177 ± 7794	2110 ± 1618	10419 ± 9295	1 ± 1	78 ± 75	20 ± 16
ADA-gelat. w/o a.i.	39963 ± 13416	0 ± 0	8309 ± 5150	48272 ± 14315	83 ± 28	0 ± 0	17 ± 11
Empty ceramic	51965 ± 31279	0 ± 0	6375 ± 2241	58340 ± 32783	89 ± 54	0 ± 0	11 ± 4

**Fig. 11:**
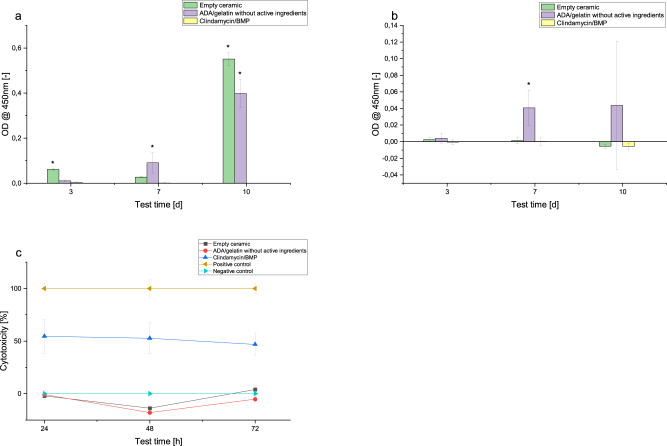
**a** Cell proliferation in the WST assay; shown as absorption at test days 3, 7 and 10. **b** Cell proliferation in the WST assay on the bottom of the respective well; shown as absorption at test days 3, 7 and 10. **c** Cytotoxicity in LDH assay after 24 h, 48 h, 72 h. (*): Result was statistically significantly different from the others of the same day

Using the WST assay, it was possible to quantify the cell proliferation that took place in the presence of the tested samples on different days. The absorption was a measure of the number of vital cells. On day 3, only the absorption of the empty ceramic was statistically significantly different from the other samples. On day 7, the same was true only for the ceramic loaded with ADA/gelatin without active ingredients. The results of the empty ceramics and those filled with ADA/gelatin without active ingredients were again significantly different from clindamycin/BMP on day 10. On this day, the major difference to the ceramics with clindamycin/BMP loading was particularly noteworthy. Here, almost no cell proliferation took place, whereas the other two samples showed strong proliferation (see Fig. 11a). For the sake of completeness, the WST results for the cells located next to the samples on the bottom of the well were shown in Fig. 11b. Here, there was statistically significant higher cell proliferation in the samples with ADA-gelatin without active ingredient compared to samples with active ingredients. Also, there was less cell proliferation in the empty ceramics samples than in samples with pure ADA-gelatin gel loaded ceramics.

The LDH assay was used to measure the cell toxicity of the tested samples. Here, 100% cell toxicity corresponded to the positive control with Triton addition, in which all cells were to be killed. All results were presented in relation to this value at the corresponding time points. 0% cell toxicity consequently corresponded to the negative control without toxic additives. The results after 24 h suggested a lack of cell toxicity of empty ceramics and loaded ceramics without active ingredients, as these values did not differ statistically significantly from the negative control. In contrast, clindamycin/BMP had a toxic effect. After 48 h, the empty ceramic and the loaded ceramic without active ingredients still did not differ statistically significantly from the negative control. The ceramics loaded with antibiotic and BMP were toxic. This result was also confirmed after 72 h (see Fig. 11c).

## Discussion

The characterisation results of the ceramics were similar to those in previous experiments [16, 17, 33, 34]. This finding was essential for comparability with the previous work. It could be proven that the produced ceramics met the specifications. Pore size and porosity influence the loading and release behaviour and have an impact on properties such as failure load and compressive strength. The molar masses of alginate and ADA were significantly lower than in another work with the same alginate used [21]. Although the molar mass was not determined by GPC but by viscosity measurement, the natural scattering range of the natural product alginate was still evident. It was remarkable that the molar mass of the alginate in another work was considerably reduced by plasma sterilisation, whereas plasma sterilisation in the present work hardly changed the molar mass of the alginate [16]. Low-temperature hydrogen peroxide gas plasma sterilisation thus proved to be a gentler process. In the literature, a 2.5% alginate gel in a similar loading procedure required a time of 10 ± 3 min for complete loading of the ceramic [14]. The rheology showed a complex viscosity of about 0.35 Pa*s at a frequency of 10 rad/s (= 1.6 Hz). At the same frequency, the complex viscosity of the ADA-gelatin gel in the present work increased during an exemplary cross-linking (measurement 1) from 0.09 Pa*s to 0.16 Pa*s after 10 min and 0.5 Pa*s after 30 min. Despite the slightly different measurement parameters, this resulted in a similar expected loading time for the ADA-gelatin gel. In comparison with another paper [21], in which both the gel production and the rheological measurement parameters matched, a significantly longer cross-linking time was shown. In Sarker et al. this was 8.2 min, in the present work the duration was at least 43 min, or crosslinking could not be accomplished at all. Despite the different properties of the alginate and the ADA produced from it, which had already been established in other studies, this large difference in the cross-linking time was remarkable. In addition, rheology verified sterile filtration as a gentle method of ADA sterilisation for cell culture experiments, but it was unusable for other experiments because of the high pressure required. In FTIR, the characteristic bands of ADA and gelatin could be shown, whereby it was noticeable in comparison to other works that the gelatin showed a peak at 1529 cm^−1^ instead of at 1543 cm^−1^ [21, 31, 35]. However, a similar band of lower wavenumber at 1522 cm^−1^ was also found in a publication by Distler et al. which made the relevance of this difference questionable [32]. In addition, evidence of the Schiff base reaction and thus the cross-linking of ADA and gelatin could be detected, which has already been described in the literature [21, 30]. In order to be able to prove the loading with the hydrogel beyond doubt, an already established procedure using FITC-protein A was used [14]. In accordance with the literature, an almost complete ceramic filling was achieved by the loading procedure with directed vacuum, whereas the procedure with undirected vacuum showed a significantly better result than described [14]. Even with the non-directional vacuum, the ceramics in the present work were completely loaded and not only in the periphery. This may have been due to the small size of the ceramics used in cell culture. On the other hand, the ADA-gelatin gel used here could have been less cross-linked at the beginning of loading and thus less viscous than the alginate sol otherwise used. Thus, loading with undirected vacuum could possibly be more suitable for ADA-gelatin gel. The recovery of clindamycin was 105 ± 5% in the microcapsule trials, which spoke for the quality of the measurement method. Since the 50% ADA and 50% gelatin gel had the lowest burst release and the highest clindamycin release per microcapsule mass on day 28, this gel composition was used for the loading trials. The measured clindamycin burst release of 43 ± 1% was lower than the vancomycin burst release of 58% in a comparable work with pure alginate microcapsules [36]. The recovery of protein A was not as high as with clindamycin in the microcapsule experiments, but still within an acceptable range. In addition, the scatter of the measurements was higher, which could be related to a certain inaccuracy due to the intrinsic fluorescence of the gelatin to be factored out, especially at the low protein A concentrations. The release from the loaded ceramics showed a comparatively high clindamycin burst release of 91 ± 3% based on the detected clindamycin mass. The clindamycin recovery was 90.8 ± 3%, almost as high as in the microcapsule trials. For BMP-2, 72 ± 53% was lost in the calcium chloride solution, which, as in known alginate experiments, led to a delayed BMP-2 release on the following days [15]. The BMP-2 recovery, on the other hand, was only in the single-digit percentage range. A possible cause could be a difference between BMP-2 and the model substance FITC-protein A, with which the loading evaluation was carried out, or a chemical interaction of BMP-2 with the gel. However, FITC-protein A has already been used as a model substance for BMP-2 in other experiments [15] and there were no indications for a chemical interaction in the literature. The fact that the release from the ceramics was less delayed than from the microcapsules was remarkable, since the ceramics should actually slow down the release further. Overall, these unexpected results were probably due to the natural variability of the alginate, which had already been noticed in the characterisation of the gels. Clindamycin release was thus comparable to that from self-crosslinking alginate [36]. The MIC for clindamycin/BMP-2 release was in the range of 0.125–0.8 µg/ml. According to EUCAST, the MIC for clindamycin in this experimental set-up should be between 0.25 and 0.5 mg/l [23]. This suggested a microbial efficacy of clindamycin until at least day 9. The increase of the MIC above the EUCAST standard on day 9 was probably due to the dilution series, whose next lower value of 0.4 mg/l was already below the upper limit of 0.5 mg/l. The MIC values below 0.25 mg/l could have resulted from a bacterial count of less than 5 x 10^5^ ml^−1^ in the inoculum control. There was thus no reason for a relevant loss of effect of the clindamycin over the release period. The good results of the RMS ceramic [14, 16, 20] and the ADA-gelatin gel [32, 37, 38] in cell culture were in line with the literature. In comparison with experiments on the release of clindamycin from pure cross-linked alginate [36], the cell experiments in this work provided less good results. Experiments in a human bone model with clindamycin release from a similar composite confirmed the negative cell culture results [39]. Possibly due to the delayed release of clindamycin from the alginate, the concentration in the cell culture was lower during the first days and thus not as toxic to the cells. On the other hand, cell survival was also not in the spirit of the experimental approach. First and foremost, the elimination of the bacteria was decisive, but the toxic effect on the cells should be tested in any case and is described here. The WST results for the cells located next to the samples on the bottom of the well showed statistically significant higher cell proliferation in the samples with ADA-gelatin without active ingredients compared to samples with active ingredients. The lack of proliferation with the antibiotics was probably due to their negative influence during diffusion into the medium. The seemingly implausible difference between empty ceramics and pure ADA-gelatin gel was explained by the fact that the cells in the medium remained in place much better on the dry empty ceramic than on a loaded ceramic. Thus, there were probably only a few cells on the well bottom in the empty ceramic and thus they could not proliferate.

**Fig. 12:**
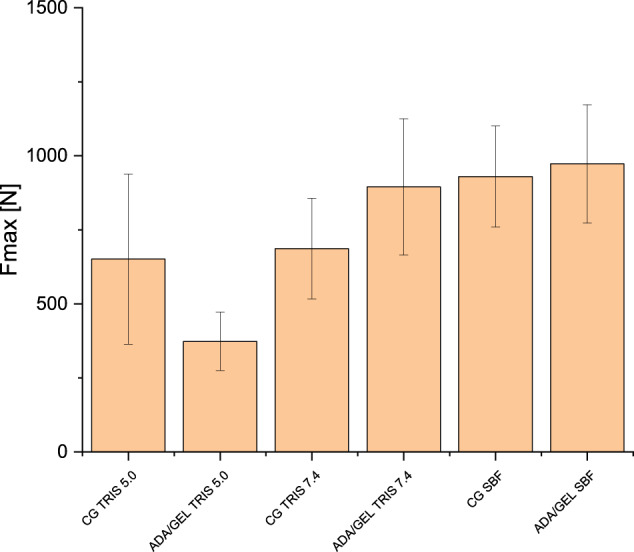
Maximum tolerated force for empty ceramics (control group, GC) and ADA-gelatin-loaded ceramics after incubation in different buffers for 30 (SBF) or 60 days

The mechanical properties of the loaded ceramics in comparison with unloaded ceramics in different buffer solutions over time were also investigated by our research group [17]. Figure 12 shows the maximum tolerated forces, for the exact measurement conditions see [17].

## Conclusions

Dual release of clindamycin and FITC-protein A from ADA-gelatin microcapsules was achieved over 28 days. The gel with equal parts of ADA and gelatin proved to be the most promising. With a clindamycin burst release of 43%, the tested gel delayed the release better than pure alginate and was also more biodegradable due to the gelatin content. The good results could not be confirmed when released from a microporous β-TCP ceramic loaded with directional flow. Nevertheless, the successful dual release of clindamycin and BMP-2 from the used composite could be demonstrated. Since the natural variability of the alginate had probably prevented complete loading, in following experiments both the necessary concentration of the alginate used should be oriented towards the rheological properties, and a more reliable ADA-gelatin cross-linking should be worked towards. Furthermore, the releases in vivo should be investigated and possible accumulations in the surrounding tissue evaluated.
